# Higher visceral adiposity index is associated with increased likelihood of abdominal aortic calcification

**DOI:** 10.1016/j.clinsp.2022.100114

**Published:** 2022-09-24

**Authors:** Zheng Qin, Luojia Jiang, Jiantong Sun, Jiwen Geng, Shanshan Chen, Qinbo Yang, Baihai Su, Ruoxi Liao

**Affiliations:** a Department of Nephrology, National Clinical Research Center for Geriatrics, Med+ Biomaterial Institute of West China School of Medicine, West China Hospital of Sichuan University, China; b Department of Nephrology, Jiujiang No. 1 People's Hospital, Jiujiang, China; c West China School of Medicine, West China Hospital, Sichuan University, China

**Keywords:** Visceral adiposity index, Abdominal aortic calcification, Vascular calcification, Cross-sectional study

## Abstract

**Background:**

The negative effects of visceral adiposity accumulation on cardiovascular health have drawn much attention. However, the association between the Visceral Adiposity Index (VAI) and Abdominal Aortic Calcification (AAC) has never been reported before. The authors aimed to investigate the association between the VAI and AAC in US adults.

**Methods:**

Cross-sectional data were derived from the 2013 to 2014 National Health and Nutrition Examination Survey (NHANES) of participants with complete data of VAI and AAC scores. Weighted multivariable regression and logistic regression analysis were conducted to explore the independent relationship between VAI and AAC. Subgroup analysis and interaction tests were also performed.

**Results:**

A total of 2958 participants were enrolled and participants in the higher VAI tertile tended to have a higher mean AAC score and prevalence of severe AAC. In the fully adjusted model, a positive association between VAI and AAC score and severe AAC was observed (β = 0.04, 95% CI 0.01‒0.08; OR = 1.04, 95% CI 1.01‒1.07). Participants in the highest VAI tertile had a 0.41-unit higher AAC score (β = 0.41, 95% CI 0.08‒0.73) and a significantly 68% higher risk of severe AAC than those in the lowest VAI tertile (OR = 1.68, 95% CI 1.04‒2.71). Subgroup analysis and interaction tests indicated that there was no dependence for the association of VAI and AAC.

**Conclusion:**

Visceral adiposity accumulation evaluated by the VAI was associated with a higher AAC score and an increased likelihood of severe AAC.

## Highlights

• Higher Adiposity Index (VAI) tertile shows higher rates of Abdominal Aortic Calcification (AAC).
• Each unit increase in VAI was associated with 4% higher likelihood of severe AAC.
• Increased visceral adiposity evaluating by VAI associated with a higher AAC score.
• This positive relationship was more significant in normal weight population.

## Introduction

Vascular Calcification (VC) is characterized by the abnormal deposition of calcium, phosphorus and other minerals in the vascular walls, which can be commonly observed in patients with Chronic Kidney Disease (CKD), diabetes, etc.^1^^,^^2^ It has been widely recognized that VC could be an important predictor of Cardiovascular Diseases (CVDs) and mortality, especially in CKD populations.^3^ There is currently no validated effective treatment with valid evidence for VC. Sodium thiosulfate and SNF472 showed their potential for alleviating the calcification progress arteries and heart valves in several small sample size randomized controlled studies.^4^, ^5^, ^6^, ^7^ However, further large-scale trials are still necessary to gain recognition for their potential use and underlying mechanisms in VC. Thus, the prevention and management of VC are of great significance for patients.^8^^,^^9^

The abdominal aorta is a common site of VC, and the presence of Abdominal Aortic Calcification (AAC) is significantly associated with both all-cause and cardiovascular mortality in patients with hemodialysis, diabetes, and even the general population.^10^, ^11^, ^12^, ^13^ To assess the severity of the calcified abdominal aorta, Kauppila et al. developed a quantitative method of AAC grading (AAC score) using lateral radiographs of the lumbar region to quantitatively evaluate the degree of calcification.^14^ A higher AAC score indicated a more severe condition of the abdominal aorta. Due to its simplicity and accuracy, the Kauppila AAC score has been applied widely in previous studies and found to independently predict all-cause mortality and cardiovascular outcomes.^15^^,^^16^

Obesity is a commonly recognized risk factor for CVDs.^17^ Body fat distribution could also be a crucial factor for cardiovascular risk; however, it is difficult to distinguish subcutaneous and visceral fat accumulation simply based on some traditional body assessment parameters, such as Body Mass Index (BMI), Waist Circumference (WC), and Waist-to-Height Ratio (WHtR).^18^ Thus, the Visceral Adiposity Index (VAI) was developed for the identification of visceral adiposity dysfunction.^19^ VAI is a novel sex-specific index based on WC, BMI, Triglycerides (TGs), and HDL Cholesterol (HDL-C), indirectly expressing visceral fat function, which has been proposed as a marker of visceral adipose tissue accumulation and dysfunction.^19^ It has also been reported to be strongly associated with cardiometabolic risks, such as hypertension, insulin resistance and increased urinary albumin excretion.^20^, ^21^, ^22^ A positive association between coronary atherosclerosis and VAI has been observed by Bagyura et al.^23^ Chen et al. found that patients with a higher VAI had more composite cardiovascular outcomes, and VAI showed a superior predictive power of composite and cardiovascular outcomes to WC and WHtR in hemodialysis patients.^24^ Previous studies have reported that elevated VAI could increase the risk of CVDs.^23^, ^24^, ^25^, ^26^ However, the relationship between VAI and AAC has not been reported before.

Thus, using data from the National Health and Nutrition Examination Survey (NHANES), the authors’ aim was to evaluate the potential associations between VAI and AAC incidence. The authors assumed that a higher VAI was associated with an increased likelihood of AAC.

## Methods

### Survey description

The authors obtained data from NHANES, a national population-based cross-sectional study to investigate nutrition and health status in the US conducted by the National Center for Health Statistics (NCHS).^27^ It was conducted with complex multistage stratified probability sampling on a biennial cycle; thus, the samples were representative.

The Research Ethics Review Board of the NCHS approved all NHANES study protocols, and written informed consent was obtained from all survey participants or a parent and/or legal guardian for participants aged below 16 years old. All detailed NHANES study designs and data are publicly available at www.cdc.gov/nchs/nhanes/.

### Study population

The present study was based on the survey cycle from NHANES 2013‒2014, since only this cycle included data on AAC score and complete variables (BMI, WC, TG, and HDL) to calculate VAI.

Participants with complete data about AAC and VAI were enrolled in the present analysis. A total of 10175 participants were enrolled at first and after the exclusion of participants aged < 40 years (they did not participate in the examination to obtain AAC score, *n* = 6360), missing the data about AAC score (*n* = 675) and VAI (total, *n* = 182; WC, *n* = 56; TG, *n* = 119; BMI, *n* = 7; HDL-C, *n* = 0), 2958 eligible participants aged ≥ 40 years were included in the final analysis (Supplemental Fig. 1).

### Definition of visceral adiposity index and abdominal aortic calcification

VAI is a sex-specific index based on WC, BMI, TG and HDL-C to estimate visceral adiposity functionality, and a higher VAI score suggested an increased amount of estimated visceral adiposity. The VAI for each participant was calculated by using the following formulas.^19^ For males: VAI = WC/(39.68+(1.88*BMI)*(TG/1.03)*(1.31/HDL-C); For females: VAI = WC/(36.58+(1.89*BMI))*(TG/0.81)*(1.52/HDL-C). TG and HDL-C were calculated in mmoL/L, and WC was calculated in cm in the formulas. VAI was treated as a continuous variable in the present analysis, and participants were grouped based on the VAI tertiles for further analysis.

The calcification severity of the abdominal aorta was represented by the AAC score. It was quantified according to the Kauppila score system by assessing lateral lumbar spine images obtained from dual-energy X-Ray absorptiometry (DXA, Densitometer Discovery A, Hologic, Marlborough, MA, USA).^15^ The total AAC score ranged from “0” to “24”, and a higher AAC score indicated more severe calcification. Based on previous studies, the authors further defined severe AAC as a total AAC score > 6, which has been widely used as a cut-off point for significant aortic calcification.^3^^,^^28^, ^29^, ^30^

In the present study, the VAI was designed as the exposure variable, and the AAC score and severe AAC were treated as outcome variables.

### Selection of covariates

Covariates in the present study included gender (male/female), age (year), race (Mexican American/other Hispanic/non-Hispanic White/non-Hispanic Black/other races), education level (less than high school, high school or general educational development/above high school), body mass index (BMI, kg/m^2^), serum creatinine (SCr, mg/dL), serum uric acid (μmoL/L), serum calcium (mmoL/L), serum phosphorus (mmoL/L), total cholesterol (mmoL/L), hypertension (yes/no) and diabetes (yes/no). Smoking status was obtained for each participant by in-home interview, and they were categorized as never, ever, current being smokers, or unknown. BMI was categorized as < 25, 25‒29.9, and ≥ 30 kg/m^2^, which corresponded to normal weight, overweight and obese populations for participants. All detailed measurement processes of these variables are publicly available at www.cdc.gov/nchs/nhanes/.

### Statistical analysis

All statistical analyses were conducted according to CDC guidelines using appropriate NHANES sampling weights and accounted for complex multistage cluster surveys. Continuous variables are summarized as the means with Standard Deviations (SDs), and categorical parameters are presented as proportions. Either a weighted Student's *t*-test (for continuous variables) or weighted Chi-Square test (for categorical variables) was employed to evaluate the differences among participants grouped by VAI tertiles. To examine the association between VAI and AAC, multivariable linear regression explored AAC score as a continuous variable, and logistic regression for severe AAC (AAC score > 6) was used as a dichotomous variable in three different models. In model 1, no covariates were adjusted. Model 2 was adjusted for sex, age and race. Model 3 was adjusted for sex, age, race, education level, body mass index, serum creatinine, serum uric acid, serum calcium, serum phosphorus, total cholesterol, hypertension, diabetes, and smoking status. Subgroup analysis of the associations of the VAI with the AAC score and severe AAC was conducted with stratified factors, including gender (male/female), age (< 60/ ≥ 60 years), BMI (normal weight/overweight/obesity), hypertension (yes/no) and diabetes (yes/no). In addition, these stratified factors were also treated as prespecified potential effect modifiers. An interaction term was added to test the heterogeneity of associations between the subgroups as well. Missing values were input by the median for continuous variables or mode for categorical variables of existing cases of those variables. All analyses were performed using R version 3.4.3 (http://www.R-project.org, The R Foundation) and Empower software (www. empowerstats.com; X&Y Solutions, Inc., Boston MA); *p* < 0.05 was considered statistically significant.

## Results

### Baseline characteristics of participants

A total of 2958 participants with an average age of 57.41±0.29 years were enrolled in this study, of whom 48.55% were male and 51.45% were female. The ranges of VAI for tertiles 1‒3 were 0.12‒1.25 (≤ 1.25), 1.25‒2.60 (≤ 2.60), and 2.60‒130.87 (≤ 130.87), respectively. The mean AAC score was 1.47±0.11 for all participants and increased with the higher VAI tertiles (Tertile 1: 1.19 ± 0.15; Tertile 2: 1.44 ± 0.11; Tertile 3: 1.77 ± 0.20, *p* = 0.0047). The prevalence of severe AAC was 7.85% overall, and participants in the higher VAI tertile tended to have higher rates of severe AAC (Tertile 1: 6.29%; Tertile 2: 7.43%; Tertile 3: 9.81%; *p* = 0.0300). Among the three VAI tertiles, differences with statistical significance were observed in race, education level, smoking status, BMI, diabetes, hypertension, serum uric acid, serum calcium, total cholesterol, HDL-C, waist circumference, and triglycerides (all *p* < 0.05). Compared with the lowest VAI group, participants in the increased VAI group were significantly more likely to have hypertension, elevated BMI, serum uric acid, serum calcium, total cholesterol, waist circumference, triglycerides, and decreased prevalence of diabetes and HDL-C levels (all *p* < 0.05). The difference between tertiles in age, sex, serum creatinine, and serum phosphorus did not meet the statistical significance (all *p* > 0.05) (Table 1).

**Table 1:** Baseline characteristics of the study population according to visceral adiposity index tertiles.

Visceral Adiposity Index	Overall	Tertile 1 (0.12‒1.25)	Tertile 2 (1.25‒2.60)	Tertile 3 (2.60‒130.87)	p-value
Age (year)	57.41 ± 0.29	56.82 ± 0.62	57.95 ± 0.45	57.48 ± 0.33	0.2916
Gender (%)					
Male	48.55 ± 0.86	48.84 ± 1.58	46.18 ± 1.59	50.60 ± 2.14	0.2650
Female	51.45 ± 0.86	51.16 ± 1.58	53.82 ± 1.59	49.40 ± 2.14	
Race (%)					
Mexican American	6.97 ± 1.63	4.55 ± 1.15	8.02 ± 1.87	8.34 ± 2.01	< 0.0001
Other Hispanic	4.68 ± 0.86	3.71 ± 0.89	5.05 ± 1.01	5.29 ± 0.89	
Non-Hispanic White	71.34 ± 3.10	71.09 ± 2.80	69.60 ± 3.59	73.31 ± 3.44	
Non-Hispanic Black	9.82 ± 1.34	14.11 ± 1.80	10.18 ± 1.59	5.19 ± 0.88	
Other Races	7.18 ± 0.77	6.54 ± 0.84	7.14 ± 1.00	7.87 ± 1.26	
Education level (%)					
Less than high school	5.02 ± 1.82	12.55 ± 1.36	14.50 ± 1.94	18.68 ± 2.76	< 0.0001
High school or GED	32.06 ± 1.44	19.11 ± 2.13	19.64 ± 1.05	26.70 ± 2.89	
Above high school	62.92 ± 2.63	68.32 ± 2.85	65.84 ± 2.54	54.61 ± 3.79	
Unknown	0.02 ± 0.01	0.03 ± 0.02	0.02 ± 0.02	0.00 ± 0.00	
Smoking status (%)					
Never	54.29 ± 1.72	60.18 ± 2.75	54.21 ± 2.21	48.49 ± 2.05	0.0001
Ever	28.41 ± 1.27	26.25 ± 2.13	29.48 ± 1.89	29.50 ± 2.04	
Current	17.30 ± 1.59	13.54 ± 2.06	16.31 ± 2.20	22.01 ± 1.50	
Unknown	0.01 ± 0.01	0.03 ± 0.03	0.00 ± 0.00	0.00 ± 0.00	
BMI (kg/m^2^)	28.53 ± 0.17	26.19 ± 0.17	28.98 ± 0.28	30.41 ± 0.24	< 0.0001
Diabetes (%)	12.94 ± 0.80	6.63 ± 0.89	11.74 ± 1.12	20.41 ± 1.55	< 0.0001
Hypertension (%)	43.58 ± 1.15	32.13 ± 2.39	45.16 ± 1.85	53.46 ± 1.70	< 0.0001
Serum creatinine (mg/dL)	0.93 ± 0.01	0.92 ± 0.01	0.92 ± 0.01	0.94 ± 0.01	0.2574
Serum uric acid (μmoL/L)	321.59 ± 1.87	301.43 ± 2.32	320.40 ± 2.07	342.89 ± 4.68	< 0.0001
Serum calcium (mmoL/L)	2.36 ± 0.00	2.36 ± 0.00	2.36 ± 0.01	2.37 ± 0.01	0.0210
Serum phosphorus (mmoL/L)	1.23 ± 0.01	1.22 ± 0.01	1.22 ± 0.01	1.23 ± 0.01	0.6103
Total cholesterol (mmoL/L)	5.05 ± 0.01	4.91 ± 0.04	4.98 ± 0.04	5.27 ± 0.03	0.0001
HDL-C (mmoL/L)	1.42 ± 0.01	1.77 ± 0.02	1.39 ± 0.02	1.08 ± 0.01	< 0.0001
Waist circumference (cm)	99.82 ± 13.57	93.16 ± 0.53	100.61 ± 0.56	105.69 ± 0.46	< 0.0001
Triglycerides (mmoL/L)	1.81 ± 0.04	0.83 ± 0.01	1.48 ± 0.02	3.11 ± 0.07	< 0.0001
AAC score	1.47 ± 0.11	1.19 ± 0.15	1.44 ± 0.11	1.77 ± 0.20	0.0047
Severe AAC (%)	7.85 ± 0.75	6.29 ± 1.04	7.43 ± 0.79	9.81 ± 1.38	0.0300

Abbreviations: GED, General Educational Development; BMI, Body Mass Index; HDL-C, High-Density Lipoprotein Cholesterol; AAC, Abdominal Aortic Calcification.

### Visceral adiposity index and increased abdominal aortic calcification

Table 2 shows the association between VAI and AAC. The present results showed that a higher VAI was associated with a higher AAC score and an increased risk of severe AAC.

**Table 2:** Association between visceral adiposity index and abdominal aortic calcification.

Visceral adiposity index groups	AAC Score	Severe AAC
	β (95% CI)	OR (95% CI)
Crude model (Model 1)		
Continuous	0.04 (-0.01, 0.09)	1.02 (1.00, 1.05)
Categories		
Tertile 1	Reference	Reference
Tertile 2	0.25 (-0.13, 0.63)	1.20 (0.83, 1.72)
Tertile 3	0.57 (0.24, 0.91)	1.62 (1.11, 2.37)
p for trend	0.0047	0.0271
Minimally adjusted model (Model 2)		
Continuous	0.05 (0.00, 0.09)	1.04 (1.02, 1.06)
Categories		
Tertile 1	Reference	Reference
Tertile 2	0.12 (-0.21, 0.44)	1.11 (0.83, 1.48)
Tertile 3	0.49 (0.16, 0.81)	1.71 (1.14, 2.57)
p for trend	0.0194	0.0371
Fully adjusted model (Model 3)		
Continuous	0.04 (0.01, 0.08)	1.04 (1.01, 1.07)
Categories		
Tertile 1	Reference	Reference
Tertile 2	0.15 (-0.17, 0.47)	1.13 (0.79, 1.60)
Tertile 3	0.41 (0.08, 0.73)	1.68 (1.04, 2.71)
p for trend	0.0138	0.0254

In sensitivity analysis, the visceral adiposity index was converted from a continuous variable to a categorical variable (tertiles).

95% CI, 95% Confidence Interval; OR, Odds Ratio; Model 1, No covariates were adjusted; Model 2, Adjusted for sex, age and race; Model 3, Adjusted for sex, age, race, education level, body mass index, serum creatinine, serum uric acid, serum calcium, serum phosphorus, total cholesterol, hypertension, diabetes and smoking status.

In the fully adjusted model, a positive association between the VAI and AAC score was observed (β = 0.04, 95% CI: 0.01‒0.08), indicating that each unit of increased VAI score was associated with 0.04 increased units of AAC score. The authors further converted the VAI from a continuous variable to a categorical variable (tertiles) to conduct the sensitivity analysis. Compared with the lowest VAI tertile, the AAC score increased with the higher VAI groups. The mean AAC score of the highest VAI tertile was 0.41 units higher than that of the lowest tertile (β = 0.41, 95% CI: 0.08‒0.73; P for trend = 0.0138) (Table 2).

For severe AAC, the authors also found a positive association between VAI and the increased likelihood of severe AAC with statistical significance. After full adjustment, subjects with a unit higher VAI had a 4% increased risk of severe AAC (Model 3: OR = 1.04, 95% CI 1.01‒1.07). The association remained statistically significant after VAI was treated as tertiles. Participants in the highest VAI tertile had a significantly 68% higher risk than those in the lowest VAI tertile (OR = 1.68, 95% CI 1.04‒2.71; P for trend = 0.0254) (Table 2).

### Subgroup analysis

**Fig. 1:**
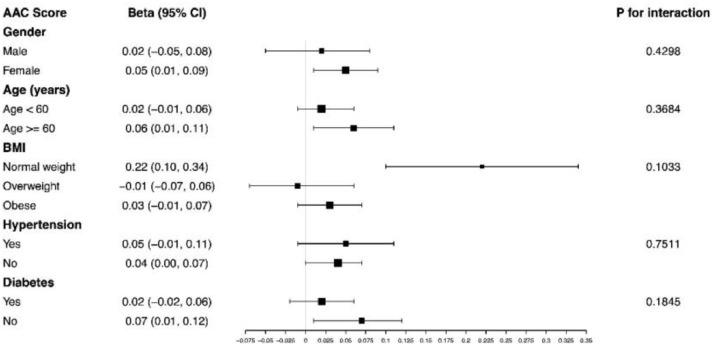
Subgroup analysis for the association between VAI and AAC score.

The present results indicated that the associations of the VAI level with the AAC score and severe AAC were not consistent. A significant relationship between VAI with AAC score was detected in females, age ≥ 60 years, normal weight, non-hypertension and non-diabetes subjects (β = 0.05, 0.06, 0.22, 0.04, 0.07, respectively) (Fig. 1).

**Fig. 2:**
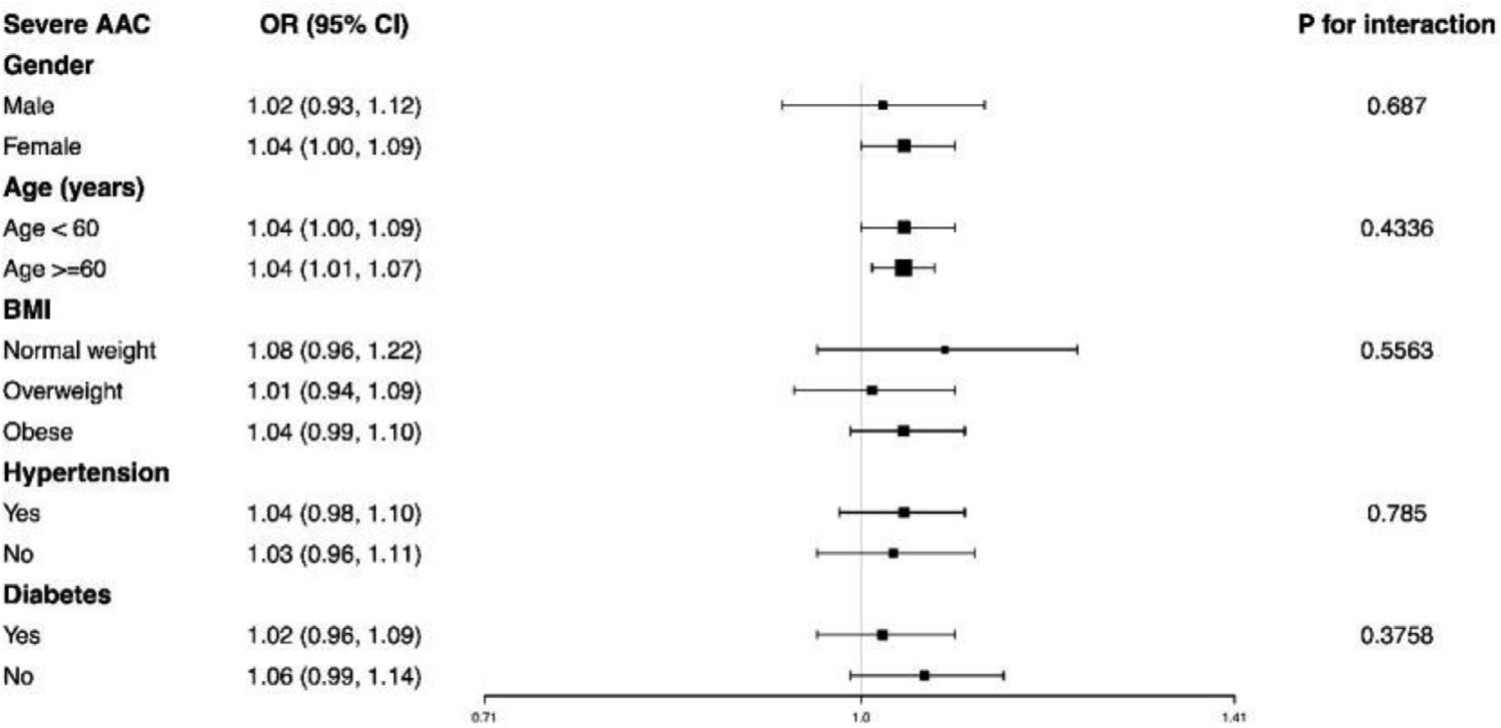
Subgroup analysis for the association between VAI and severe AAC.

For the association between VAI and severe AAC, the authors observed a positive association in females and participants stratified by age less than 60 years or not. Each unit increase in VAI was associated with 4% higher likelihood of severe AAC both in those aged less than 60 years (OR = 1.04, 95% CI 1.00‒1.09) and more than 60 years (OR = 1.04, 95% CI 1.01‒1.07). The interaction term did not report the influence of age on the association between VAI and AAC (P for interactio*n* = 0.4336). In addition, there was no significant difference suggested by the interaction test in the association of VAI with AAC score and severe AAC among different stratifications, indicating that there was no significant dependence of gender, age, BMI, hypertension, and diabetes on this positive association (all p for interaction > 0.05) (Fig. 2).

## Discussion

In the cross-sectional study that enrolled 2958 participants, the authors observed a positive association between the VAI and AAC, and there was no significant dependence of sex, age, BMI, hypertension, or diabetes on this association, indicating that an increased VAI may contribute to a higher AAC score and an increased risk of severe AAC. The present results indicated that the management of visceral fat distribution may alleviate the occurrence of AAC.

To our knowledge, this is the first study assessing an association between VAI and AAC. Previous studies have explored the relationship between VAI and CVDs. Chen et al. performed a prospective study including 464 prevalent hemodialysis patients and found that patients with a higher VAI showed an increased risk of composite cardiovascular outcomes and all-cause death.^24^ Bagyura et al. conducted a cross-sectional study with 460 participants and observed that a higher VAI tertile could be an independent predictor of the presence of coronary atherosclerosis.^23^ Amato et al. also reported that VAI was independently associated with both cardiovascular and cerebrovascular events, suggesting that AVI could be a valuable indicator of cardiometabolic risk.^19^ In a large-sample, long-term, prospective study in Europe, Kouli et al. reported that VAI was independently associated with elevated 10-year CVD risk, particularly in men, which suggested that VAI may be utilized as an additional indicator of long-term cardiovascular outcome risk for Caucasian/Mediterranean individuals.^25^ Yang et al. found that the VAI was positively associated with hypertension among the Chinese adult population, and it may be an indicator of hypertension risk for the Chinese population.^26^ Consistent with the negative effects of higher VAI on cardiovascular health reported by previous studies, the authors also observed a positive association between VAI and increased likelihood of calcified abdominal aorta, supporting the intense association between VAI and cardiometabolic risks. Considering that VAI is an optimal method to measure visceral adiposity, the present results indicated that the management of visceral fat distribution may alleviate the process of vascular calcification.

Visceral obesity is a marker of dysfunctional adipose tissue and a well-known risk factor for CVDs.^17^ WC and WHtR have been used widely in previous studies to evaluate the degree of abdominal adiposity, and they are recognized as the gold standard for visceral adiposity measures.^31^ Postorino et al. found that higher WC and WHtR were direct predictors of all-cause and CV mortality in patients with end-stage renal disease, suggesting that abdominal obesity underlies an increased risk of poor prognosis.^32^ Sanches et al. also observed a strong relationship between WC and visceral fat in CKD patients, and the associations between WC and CVD risk factors were similar to those observed for visceral fat, which suggested that WC may be a simple and inexpensive tool to indicate visceral adiposity.^33^ However, WC cannot distinguish visceral and subcutaneous fat in the abdominal region and represents them together, which may lead to controversial outcomes.^34^, ^35^, ^36^ In addition, the predictive power of WC for CVD was adjusted for BMI; thus, an interaction between WC and BMI may influence the outcomes.^32^ A similar influence has also been reported to be affected by the interaction between WC and triglycerides and adipokines.^37^^,^^38^ According to the recommendation from the International Diabetes Federation, Magnetic Resonance Imaging (MRI) and Computed Tomography (CT) are precise and reliable.^39^ However, these machine-based measurements are costly and complicated to conduct for some individuals, such as patients with dialysis and CKD. Thus, using VAI, a mathematical model including both anthropometric and metabolic parameters, to evaluate the adipose distribution of patients may be a better tool for assessing the impacts of visceral adiposity on cardiovascular outcomes. In the fully adjusted model, VAI Tertile 3 showed a higher AAC score (β = 0.41, 95% CI 0.08‒0.73) and the likelihood of severe AAC (OR = 1.68, 95% CI 1.04‒2.71) than Tertile 1, indicating the negative effect of visceral adiposity on VC. The present results of subgroup analysis stratified by BMI found that this positive association was significant in the normal-weight population, indicating that even for normal-weight participants, higher visceral fat was associated with an increased risk of aortic calcification. However, the interaction test demonstrated that there was no dependence of sex, age, BMI, hypertension, or diabetes on this positive association between VAI and AAC (all p for interaction > 0.05), suggesting that these positive correlations were similar in different populations settings. The present results supplemented and confirmed the negative effect of visceral adiposity on cardiovascular health in a general population.

This study has several strengths. First, this study was based on the data from NHANES, which is nationwide, population-based sampling data obtained using a standard protocol. All analyses were performed with consideration of appropriate NHANES sampling weights, making the study samples more representative. The authors also adjusted for confounding covariates to ensure that the present results were more reliable. However, the limitations of this study cannot be ignored. The authors cannot obtain a clear causal relationship due to the cross-sectional study design. In addition, it was noted that the VAI measurement was analyzed in a timely manner in this study and this information may not reflect the long-term reality of these patients. While the aortic calcification is a more perennial data, data over time in relation to VAI could be more useful for this topic, thus, a subsequent large-scale cohort study may be necessary to further confirm the present results. Although the results were based on a national representative dataset, the data the authors utilized was obtained from 2013‒2014, which is about ten years ago. The authors tried to use more recent data to analyze the association, but data about AAC score was only available in NHANES 2013‒2014 and other NHANES survey cycles did not collect the information about AAC. Second, although some potential covariates have been adjusted, the authors cannot completely exclude the effect of other possible confounding factors, for example, the use of drugs, some other comorbidities including aortic aneurysm or ectasia, etc. These data were not available in the NHANES study design, which may affect the present data interpretation. In addition, due to the NHANES study design, participants aged less than 40 years did not receive dual-energy X-Ray absorptiometry, and their AAC score data were missing; thus, the authors could not further explore the relationship between VAI and AAC for a wide age group.

## Conclusion

The present study demonstrated that elevated VAI levels were associated with higher AAC scores and an increased likelihood of severe AAC. The present findings highlight the importance of the management of visceral adipose accumulation in identifying patients at risk of AAC. However, further large-scale prospective studies are still needed to validate the authors’ findings.

## Data availability statement

Publicly available datasets were analyzed in this study. These data can be found at: www.cdc.gov/nchs/nhanes/.

## Ethics statement

The studies involving human participants were reviewed and approved by the National Center for Health Statistics. The patients/participants provided written informed consent to participate in this study.

## Authors' contributions

Zheng Qin: Investigation, methodology, writing-original draft preparation; Luojia Jiang: Software, writing-original draft preparation; Jiantong Sun: Data curation; Jiwen Geng: Methodology; Shanshan Chen: Validation; Qinbo Yang: Supervision; Baihai Su: Methodology; Ruoxi Liao: Conceptualization, writing-reviewing and editing.

## Declaration of Competing Interest

The authors declare no conflicts of interest.

## Appendix Supplementary materials

Image, application 1

## Footnotes

Supplementary material associated with this article can be found, in the online version, at doi:10.1016/j.clinsp.2022.100114.
