# Supplementary material for: Higher visceral adiposity index is associated with increased likelihood of abdominal aortic calcification
Source: Clinics (Sao Paulo). 2022 Sep 24;77:100114. doi: 10.1016/j.clinsp.2022.100114 (PMC9513216; doi:10.1016/j.clinsp.2022.100114)

**CLINICS-D-22-00268 - Supplementary Material**

**Supplemental Figure 1** Flowchart of the sample selection from NHANES 2013‒2014.


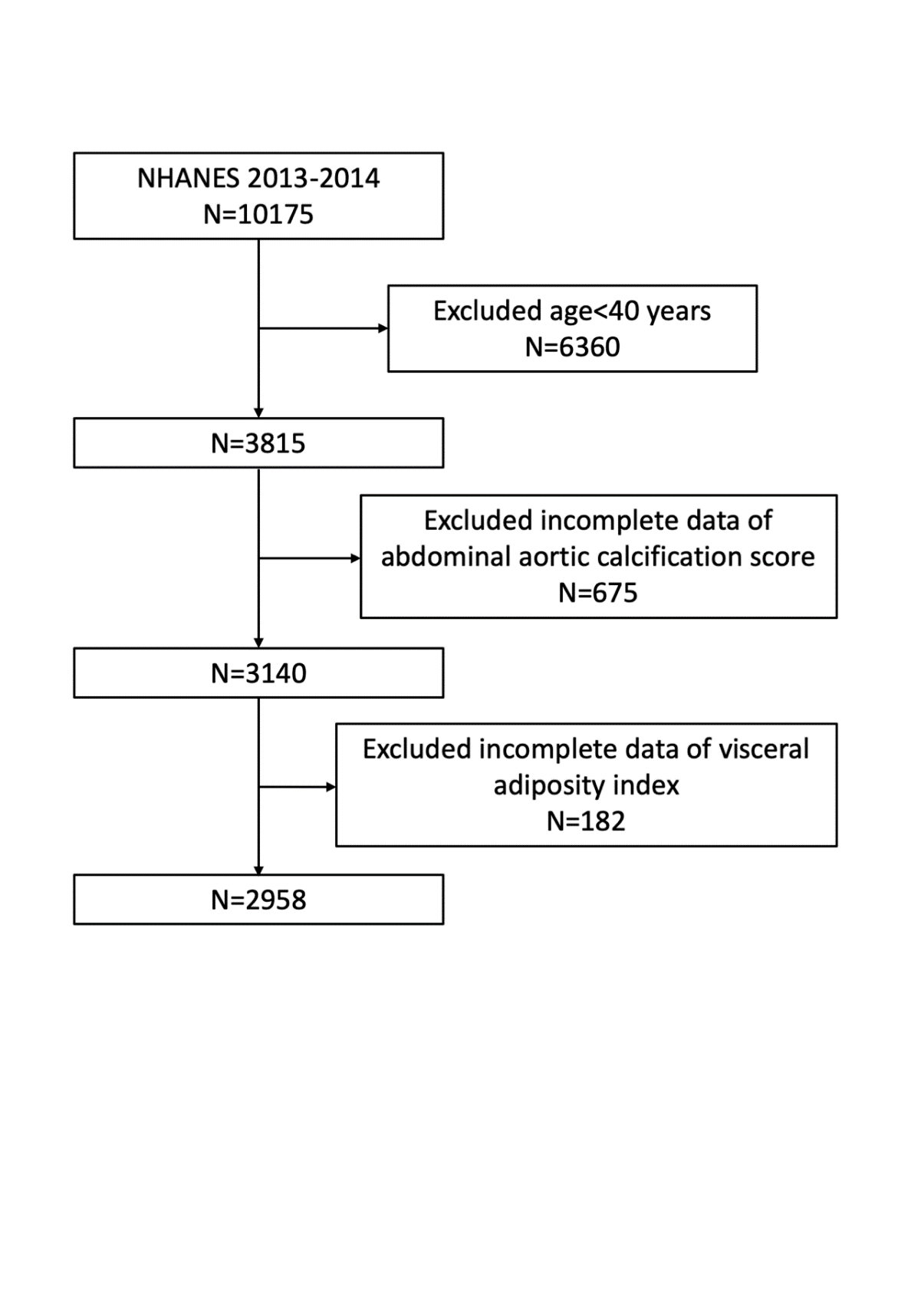

Supplement: Supplementary file 1 [file mmc1.docx]
